# The Association between Apolipoprotein E Gene Polymorphism and Mild Cognitive Impairment among Different Ethnic Minority Groups in China

**DOI:** 10.1155/2014/150628

**Published:** 2014-08-05

**Authors:** ZhiZhong Wang, Wanrui Ma, Ye Rong, Lan Liu

**Affiliations:** ^1^Department of Epidemiology and Biostatistics, School of Public Health, Ningxia Medical University, Yinchuan 750004, China; ^2^Department of Comprehensive Medicine, General Hospital of Ningxia Medical University, Yinchuan 750004, China; ^3^Brain and Mind Research Institute, University of Sydney, Sydney, NSW 2050, Australia

## Abstract

The association, in different ethnic groups, of apolipoprotein E (apoE) gene polymorphism with mild cognitive impairment (MCI) has been unclear. Few studies have examined the association in Chinese minorities. The current study explores the association between apoE gene polymorphism and MCI in one of the biggest ethnic groups—the Hui—and compares it with the Han. The Minimental State Exam, Activities of Daily Living Scale, and Geriatric Depression Scale were administered to 306 ethnic Hui and 618 ethnic Han people aged ≥55 years. ApoE genotypes were determined using the high resolution melting curve method. The distribution of the apoE genotype and the frequency of alleles *ε*2, *ε*3, and *ε*4 were similar in the Hui and Han groups. In analyses adjusted for age, gender, and education level, the *ε*4 allele was a risk factor for MCI in both the Hui group (OR = 2.61, 95% CI: 1.02–6.66) and the Han group (OR = 2.36, 95% CI: 1.19–4.67), but the apoE *ε*2 allele was protective for MCI only in the Han group (OR = 0.48, 95% CI: 0.38–0.88). The association of some apoE genotypes with MCI may differ in different ethnic groups in China. Further studies are needed to explore this effect among different populations.

## 1. Introduction

Apolipoprotein E (apoE) is a plasma protein involved in regulating the body's metabolism of lipoproteins and cholesterol balance. It has an important role in the neurobiological system [1]. Human apoE has three isoforms, *ε*3, *ε*4, and *ε*2, due to the cysteine-arginine interchanges at codons 112 and 158 [2]. The most frequently occurring allele is *ε*3, followed by *ε*4 and *ε*2 [1].

The apoE *ε*4 allele has been widely studied as a risk factor for mild cognitive impairment (MCI) and Alzheimer's disease (AD), but its impact has been found to differ across ethnic groups [3–7]. Seet et al. reviewing the frequency of the apoE alleles in different ethnic groups found that the relative frequency of *ε*4 was the lowest in the Chinese sample [8]. This raised a question as to whether the Chinese people did in fact benefit from the reduced frequency of the apoE *ε*4 allele. The prevalence of MCI was estimated to be over 18.5% among Chinese people aged 55 years or older [9], with about seven million Chinese people suffering from AD among people aged 55 years or older [10].

Within the Chinese population, studies have shown that apoE *ε*4 increases the risk of MCI in the elderly Han ethnic group (the majority group) [11, 12], but few studies have examined the association between the *ε*4 allele and MCI among different ethnic minority groups in China, although the frequency of apoE genotypes has been found to differ across different ethnic groups [13–15]. China has fifty-five national minorities, among which the Hui ethnic group is the largest minority group. The Chinese Hui ethnic group has descended from Arab and Persian Muslim immigrants [16]. They share the same language, customs, and living environments with the local Han majority but are different in terms of genetic background [17]. Deng et al. investigated the diversity distributions of 15 short tandem repeats (STRs) loci in a sample of the Hui ethnic group comparing them with other Chinese ethnic groups. The results showed a significant difference between the Hui and other ethnic groups in some loci [18]. In addition, previous studies of community residents showed that the Hui ethnic group had a higher prevalence of abnormal lipid metabolism than the local Han population [19]. However, there is no study focused on the frequency of the apoE *ε*4 allele and the association between the *ε*4 allele and MCI in the Hui ethnic minority.

In the current study, we aim to compare the relative frequency of the apoE alleles *ε*2, *ε*3, and *ε*4 in a community-based sample of the Hui ethnic group with a comparable sample of the Han ethnic group in Mainland China and to examine the association between apoE gene polymorphism and MCI in those two groups.

## 2. Materials and Methods

### 2.1. Subjects

Five communities were selected from two cities using a convenience cluster sampling method. Among them, three communities were from Yinchuan (the biggest city in the Ningxia province, and over 80% of the population is Han ethnicity) and two communities were from Wu Zhong (the second biggest city in the Ningxia province, and over 80% of the population is Hui ethnicity). Individuals aged 55 years or older who had permanent residency and agreed to participate were included in the study. Those who could not complete the survey due to vision and hearing disabilities, a long history of alcohol consumption, or serious diseases were excluded. Demographic information was collected using a questionnaire designed by the research team, and the ethnic identity was verified by residency registration information (called* hukou* in Chinese) authorized by the local government. The residency registration system is the basic institution which documents population information and distributes public resources in China [20].

Of 1022 participants enrolled in the study, 924 (90.5%) completed the entire examination, including 306 Hui ethnic people (mean age is 65.4 years; standard deviation (SD) is 6.8) and 618 Han ethnic people (mean age is 66.9 years; SD is 6.5).

This study was approved by the Institutional Review Board of the Ningxia Medical University. Participants provided written informed consent prior to the survey.

### 2.2. Neuropsychological Testing and Physical Examination

All the participants had a face-to-face interview performed by trained medical students using a structured questionnaire, which included questions on smoking, alcohol use, memory, and medical history. A series of tests including the Minimental State Exam (MMSE) [21], Activities of Daily Living Scale (ADL) [22], and the Geriatric Depression Scale (GDS) [23] were administered by two clinicians from the Geriatrics Department of Ningxia Medical University. MCI was diagnosed according to Peterson's criteria [24]. One hundred and eighty-one people (19.6%) met the criteria for MCI, including 66 Hui and 115 Han ethnic people (Table 1). All participants underwent a careful physical examination in community health care centers to obtain the blood pressure and disease history.

### 2.3. ApoE Gene Polymorphism Test

Genomic DNA was isolated from venous blood leukocytes using a genomic DNA extraction and purification kit following the manufacturer's protocol (Wizard, Genomic DNA Purification Kit; Promega Company, USA). The primer was made by Invitrogen Company and diluted and kept at −20°C. An 89 bp PCR product encompassing acid position rs429835 (112) was produced by using the forward primer 5′-CGGGCACGGCTGTCCAAG-3′ and the reverse primer 5′-CGGTACTGCACCAGGCGGC-3′. A 70 bp PCR product encompassing acid position rs7412 (158) was produced by using the forward primer 5′-GCTGCGTAAGCGGCTCCTCC-3′ and the reverse primer 5′-GGCCCCGGCCTGGTACACT-3′.

ApoE gene polymorphism was detected by the high resolution melting (HRM) curve method [25]. Instruments were undertaken in a Roche Light Cycler 480 (Roche, USA). The reaction system was composed of 5 *μ*L reaction mixture, 0.5 *μ*L primer mix, 0.5 *μ*L fluorescein EVA, and 3 *μ*L pure water. The total volume was 9 *μ*L. The system was placed in a 1.5 mL Eppendorf tube and then in a vortex momentarily for mixing. The reaction system was added to Roche 96 pore plates, and each pore plate was added 1 *μ*L DNA moldboard. A computerized test was run after shocking. All detections were completed by professional technicians at the Biochip Ningxia Center following the manufacturer's instructions. In order to control for an accurate genotype call, twelve DNA samples were retested using the dideoxy-mediated chain-termination method in an independent Lab (Genex, China). All the genotypes were detected by the sequencing method consistent with that detected by the HRM.

### 2.4. Statistical Analyses

We identified the carriers of the *ε*2 allele, including genotypes *ε*2*ε*2 and *ε*2*ε*3, and the carriers of the *ε*4 allele, including genotypes *ε*3*ε*4 and *ε*4*ε*4, and *ε*3*ε*3 as control. A gene risk index model, widely used in other studies, was used to evaluate the variation in apoE polymorphisms [26]. The model assigned +1, 0, or −1 for *ε*2, *ε*3, or *ε*4 allele, respectively. The genotypes *ε*2*ε*2, *ε*2*ε*3, *ε*2*ε*4, *ε*3*ε*3, *ε*3*ε*4, and *ε*4*ε*4 then had scores of +2, +1, 0, 0, −1, and −2, respectively. The distributions of apoE genotypes, the frequency of the alleles (*ε*2, *ε*3, and *ε*4), *ε*2 allele carrier, *ε*4 allele carrier, and demographical variables between Han and Hui or between MCI and no MCI were analyzed using the R ∗ C chi-square test. Age and apoE risk index were analyzed using Student's *t*-test. Logistic regression model was performed to obtain the odds ratios (OR) and 95% confidence intervals (CI) for the *ε*2 or *ε*4 allele carriers after controlling for age, gender, and level of education. Also, two separated logistic regression models performed stratified by ethnic. The statistically significant level was set at *P* < 0.05. All analyses were performed using the statistical package for social sciences version 16.0 (SPSS Inc., Chicago, IL, USA).

## 3. Results 

### 3.1. Frequency of ApoE Allele and Genotype Distributions in the Hui and Han Ethnic Groups

There was no statistically significant difference in the distribution of the six kinds of genotypes and three kinds of allele between the Hui and Han ethnic groups (Table 1). Compared with the Han ethnic group, the Hui ethnic group had higher levels of serum total cholesterol and diastolic blood pressure and lower proportions of smoking, alcohol use, and living alone and lower education level.

### 3.2. The Bivariate Association between ApoE *ε*4 Allele and MCI

Table 2 shows the association between apoE *ε*4 allele and MCI; we investigated the association between the apoE *ε*4 allele and MCI in the Hui and Han ethnic groups combined and separately. Consistent correlations between apoE *ε*4 allele and MCI were found in three subgroups. The proportion with the *ε*4 allele (7.5% versus 3.2%) and of *ε*4 carriers (14.4% versus 5.7) was significantly higher in the participants with MCI than those without MCI; *P* < 0.001. There was a significantly lower risk index score for those with MCI in the combined group (*P* = 0.002) and for the Han ethnic group (*P* = 0.007), but not in the Hui ethnic group (*P* = 0.118).

### 3.3. Logistic Regression Analysis for ApoE *ε*4 Allele and MCI (Table 3)

After controlling for age, gender, and education level, the *ε*4 carrier was a risk factor for MCI in both the Hui (OR = 2.61) and the Han (OR = 2.36) ethnic groups. The *ε*2 carrier was protective for MCI in the Han ethnic group (OR = 0.48), but not in the Hui ethnic group.

## 4. Discussion

The present study found that there was no significant difference in apoE genotype and *ε*4 allele frequency between older Hui and Han ethnic groups. This finding is similar to that for the Chinese Uyghur ethnic people [15]. However, compared with the Saudi Arabia Muslims who are believed to be the ancestors of the Chinese Hui ethnic people, the frequency of *ε*4 allele in the Hui ethnic group (3.9%) was close to that in the Arabs (3.9–6.3%) [27, 28].

The frequency of the *ε*4 allele is 4.4% in Hui and 3.9% in Han in current study; this finding agrees with previous studies which have reported that the frequency of apoE genotypes is significantly different in different races and ethnicities. The gene frequency of *ε*4 allele was from 12.4% to 19.8% in Caucasians, while the Chinese Han ethnic population has been found to have a lower frequency of the *ε*4 allele, ranging from 7.5% to 8.7% [8, 13, 26]. Regarding other Chinese minorities, the frequency of *ε*4 allele ranged from 4.9% in the Zhuang ethnic group [29] to 15.4% in the Mongolian ethnic group [30].

Studies have shown that apoE *ε*4 carrier increases the risk of MCI in the elderly Chinese Han ethnic group [11, 12]. However, except for the influence of gender, age, and level of education, current study found a consistent association between the *ε*4 allele and MCI in both the Han and the Hui ethnic groups, and these findings were similar to that of other studies [31]. MCI was the most commonly accepted prodromal AD stage; this suggests that the apoE *ε*4 allele has relevance for MCI screening [32, 33].

In contrast, apoE *ε*2 allele has been reported to be a protective factor for cognitive function in elderly people. Presence of the apoE *ε*2 allele was reported to have an association with improvement in episodic memory over time [34] and to reduce the risk of cognitive decline among older adults [35]. A recent study showed no significant association between apoE *ε*2 presence and MCI in Chinese Han ethnic people, but apoE *ε*2 was inversely associated with AD [11]. The current study found that the *ε*2 carrier was a protective factor for MCI in the Han ethnic group, but not in the Hui ethnic group. This difference may be due to the relatively smaller sample size for Hui participants and the relatively lower frequency of *ε*2 in Chinese population which reduced statistical power [26]. For the present study, the power for logistic model was 0.85 in Han groups and 0.73 in Hui groups, respectively.

## 5. Conclusions

In summary, this study has shown that apoE *ε*4 was a risk factor for MCI in both of the Han and Hui ethnic groups in China. But the association of apoE *ε*2 with MCI may differ in different ethnic groups in China. Further studies are needed to explore this effect among different populations.

## 6. Limitations

There are several limitations to the present study. First, the cross-sectional design of this study could only detect the association between genes and MCI, but not a causal relationship. Second, the samples were not randomly selected so that they could not represent the general population. In addition, the sample size of the Hui ethnic group was relatively smaller than that of the Han ethnic group, which might reduce the comparability of the two samples.

## Figures and Tables

**Table 1:** Characteristics of the studied population.

	Total (*N* = 924)	Hui (*N* = 306)	Han (*N* = 618)	*P* value∗
Age, mean (SD), and years	66.4 (6.6)	65.4 (6.8)	66.9 (6.5)	0.001
Gender, female, *n* (%)	571 (61.8)	183 (59.8)	388 (62.7)	0.380
Education, *n* (%)				
No formal education	239 (25.9)	106 (34.6)	133 (21.5)	<0.001
Primary school	253 (27.4)	89 (29.1)	164 (26.5)	
Junior high school	254 (27.5)	69 (22.5)	185 (29.9)	
Senior high school or above	178 (19.3)	42 (13.7)	136 (22.0)	
Living arrangement, alone, *n* (%)	187 (20.2)	48 (15.7)	139 (22.5)	0.015
Smoking, yes, *n* (%)	218 (23.6)	48 (15.6)	170 (27.5)	<0.001
Alcohol use, yes, *n* (%)	115 (12.4)	14 (4.5)	101 (16.3)	<0.001
TC mean (SD) (mmol/L)	4.7 (0.9)	4.6 (1.0)	4.8 (0.9)	0.051
TG mean (SD) (mmol/L)	1.8 (0.8)	1.7 (0.9)	1.8 (0.8)	0.273
HDL mean (SD) (mmol/L)	4.0 (1.0)	4.0 (1.0)	4.0 (1.0)	0.738
LDL mean (SD) (mmol/L)	3.2 (0.6)	3.2 (0.7)	3.2 (0.6)	0.307
SBP mean (SD) (mmHg)	149.7 (20.4)	151.2 (20.9)	148.9 (20.2)	0.104
DBP mean (SD) (mmHg)	87.0 (11.5)	88.5 (11.4)	86.3 (11.4)	0.005
MCI, yes, *n* (%)	181 (19.6)	66 (21.5)	115 (18.6)	0.286
apoE genotype *n* (%)				
*ε*2/2	19 (2.1)	8 (2.6)	11 (1.8)	0.517
*ε*2/3	122 (13.2)	39 (12.4)	83 (13.4)	
*ε*2/4	3 (0.3)	2 (0.7)	1 (0.2)	
*ε*3/3	715 (77.4)	237 (77.2)	479 (77.5)	
*ε*3/4	58 (6.3)	17 (5.5)	41 (6.6)	
*ε*4/4	7 (0.8)	4 (1.3)	3 (0.5)	
apoE allele frequency, *n* (%)				
*ε*2	163 (8.8)	57 (9.3)	106 (8.6)	0.752
*ε*3	1,612 (87.1)	530 (86.3)	1082 (87.5)	
*ε*4	75 (4.0)	27 (4.4)	48 (3.9)	
*ε*4 carrier, yes, *n* (%)	68 (7.4)	23 (7.5)	45 (7.2)	0.898
*ε*2 carrier, yes, *n* (%)	144 (15.6)	49 (16.0)	95 (15.4)	0.800
Risk index, mean (SD)	0.1 (0.5)	0.1 (0.5)	0.1 (0.5)	0.913

*P value of Hui with Han; TC: total cholesterol; TG: triglyceride; HDL: high density lipoprotein; LDL: low density lipoprotein; SBP: systolic blood pressure; DBP: diastolic blood pressure.

**Table 2:** Bivariate association between apoE *ε*4 allele and MCI.

Total (*n* = 924)	MCI		*P* value
	NO (*N* = 743)	Yes (*N* = 181)	
Allele frequency, *n* (%)			
*ε*2	139 (9.4)	24 (6.6)	<0.001
*ε*3	1299 (87.4)	311 (85.9)	
*ε*4	48 (3.2)	27 (7.5)	
*ε*2 carrier, yes, *n* (%)	123 (16.6)	21 (11.6)	0.100
*ε*4 carrier, yes, *n* (%)	42 (5.7)	26 (14.4)	<0.001
Risk index, mean (SD)	0.1 (0.5)	−0.0 (0.5)	0.002

Hui (*n* = 306)	NO (*N* = 240)	Yes (*N* = 66)	

Allele frequency, *n* (%)			
*ε*2	46 (9.6)	11 (8.3)	<0.001
*ε*3	418 (87.1)	110 (83.3)	
*ε*4	16 (3.3)	11 (8.3)	
*ε*2 carrier, yes, *n* (%)	39 (16.25)	10 (15.1)	0.829
*ε*4 carrier, yes, *n* (%)	13 (5.4)	10 (15.1)	0.008
Risk index, mean (SD)	0.1 (0.5)	0.0 (0.6)	0.118

Han (*n* = 618)	NO (*N* = 503)	Yes (*N* = 115)	

Allele frequency (%)			
*ε*2	93 (9.2)	13 (5.7)	<0.001
*ε*3	881 (87.6)	201 (87.4)	
*ε*4	32 (3.2)	16 (7.0)	
*ε*2 carrier, yes, *n* (%)	84 (16.6)	11 (9.5)	0.056
*ε*4 carrier, yes, *n* (%)	29 (5.7)	16 (13.9)	0.002
Risk index, mean (SD)	0.1 (0.5)	0.0 (0.5)	0.007

**Table 3:** Logistic regression of apoE gene polymorphism and MCI.

	*β*	SE	Wald *χ* ^2^	*P*	OR	95% CI
Total (*n* = 924)						
ethnicity	−0.33	0.18	3.39	0.066	0.71	0.49–1.02
Risk index	−0.46	0.16	7.80	0.005	0.63	0.45–0.87
*ε*2 carrier	−0.38	0.27	1.99	0.158	0.68	0.39–1.16
*ε*4 carrier	0.89	0.28	9.93	0.002	2.45	1.40–4.29
Hui (*n* = 306)						
Risk index	−0.53	0.26	0.74	0.389	0.79	0.47–1.33
*ε*2 carrier	0.10	0.41	0.06	0.807	1.10	0.49–2.47
*ε*4 carrier	0.96	0.47	4.04	0.044	2.61	1.02–6.66
Han (*n* = 618)						
Risk index	−0.53	0.21	6.36	0.012	0.58	0.38–0.88
*ε*2 carrier	−0.71	0.34	4.20	0.040	0.48	0.24–0.96
*ε*4 carrier	0.86	0.34	6.16	0.013	2.36	1.19–4.67
